# Three-dimensional cellular visualization in mouse apical periodontitis using combined whole-mount staining and optical tissue clearing

**DOI:** 10.1016/j.job.2022.12.003

**Published:** 2022-12-29

**Authors:** Kento Tazawa, Hajime Sasaki

**Affiliations:** a Department of Cariology, Restorative Sciences, and Endodontics, University of Michigan School of Dentistry, 1011 N University Avenue, Ann Arbor, MI, 48109, USA; b Department of Pulp Biology and Endodontics, Division of Oral Health Sciences, Graduate School of Medical and Dental Sciences, Tokyo Medical and Dental University (TMDU), Tokyo, Japan

**Keywords:** Staining and labeling, Three-dimensional imaging, Periapical periodontitis, Fluorescent dyes

## Abstract

Apical periodontitis is an inflammatory disease involving lesions located within the jawbone. Histological evaluations generally require decalcification and sectioning, which has limited our understanding of the three-dimensional (3D) organization and spatial distribution of different immune cell types in these lesions. A recently developed technique combining tissue clearing and whole-mount immunofluorescent labeling allows us to acquire such information from the deep tissue without sectioning. However, whole-mount immunofluorescent labeling in the jawbone requires further development. Here we provide a straightforward and efficient protocol to achieve 3D immunofluorescent imaging of murine periapical lesions.

## 1. Introduction

Apical periodontitis is an inflammatory disease subsequent to pulp infection with bone destruction and infiltration of various immune cells into the lesion [1]. Although histological evaluation of apical periodontitis has been evaluated [2–4], researchers have yet to obtain detailed information regarding the three-dimensional (3D) organization and spatial distribution of immune cell types. One of the critical factors increasing the difficulty of 3D imaging of periapical lesions is that the lesions occur inside the jawbone, requiring demineralization and sectioning for histological evaluation, and observations are restricted to a two-dimensional (2D) view. Although 3D reconstruction based on serial sections throughout periapical lesions is a traditional approach to obtaining such 3D data [5–7], serial sectioning is highly technique-sensitive and time-consuming.

Researchers have recently developed various tissue clearing techniques that allow 3D visualization of deep tissue [8]. 3D visualization usually requires labeling targets with bright fluorescent tags, and transgenic mouse strains expressing fluorescent proteins are invaluable tools for this purpose due to their labeling reliability and simplicity. However, multiple mutations are required according to the number of target molecules, and labeling multiple molecules is technically challenging. Antibody-based immunofluorescent labeling is a reliable alternative for labeling the target molecule without the genetic mutation. However, whole-mount immunofluorescent labeling has not been well established in deep tissues surrounded by hard tissue (e.g., the periapical lesion and the dental pulp), where it is difficult to achieve adequate antibody penetration. Therefore, it is essential to develop a reliable whole-mount immunolabeling method.

Here, we have established a whole-mount staining technique for mandible specimens. Combined with the polyethylene glycol-associated solvent system (PEGASOS) method [9], this technique facilitates the acquisition of spatial distribution data of different target cells in periapical lesions. In this report, we present the details of this technique and demonstrate its advantages over serial histopathological microscopic images.

## 2. Reagents and solutions

- Primary antibodies: Rat anti-mac2 conjugated biotin (#125403, Biolegend, San Diego, CA) and Rabbit anti-arginase1 (Arg1) (#16001–1-AP, Proteintech, Rosemont, IL).
- Secondary antibodies: Alexa Fluor 488 conjugated goat anti-rabbit (#ab150077, Abcam, Cambridge, MA) and Alexa Fluor 594 conjugated goat anti-rat (#ab150160, Abcam).
- 10 mM HEPES (staining buffer): Dissolve 10% (v/v) Triton X-100 (#T8787, Sigma-Aldrich, Burlington, MA), 200 mM sodium chloride (NaCl, #S9888, Sigma-Aldrich), 0.5% goat serum, and 0.05% sodium azide (#S2002, Sigma-Aldrich) in deionized distilled water (DDW).
- Blocking buffer: Staining buffer containing 10% goat serum.
- 4% paraformaldehyde (PFA) solution (pH 7.4): 2 g of PFA powder (#158127, Sigma-Aldrich) is dissolved in 40 ml of preheated DDW at 60 °C. Then add 8 μl of 5 N sodium hydroxide (NaOH, #SS256–500, Thermo Fisher Scientific, Waltham, MA) and 5 ml of 10x PBS. Lastly, add DDW to bring the final volume to 50 ml.
- 15% ethylenediaminetetraacetic acid (EDTA) (pH 8): 75 g of EDTA powder (E9884, Sigma-Aldrich) and 20 g of NaOH (#567530, Sigma-Aldrich) are dissolved in 400 ml of DDW. Then, add 5 N NaOH solution (Thermo Fisher Scientific) until the solution is pH 8. Lastly, add DDW to bring the final volume to 500 ml.
- Tris-buffered saline with Triton X-100 (TBST) (pH 7.4): Dissolve 50 mM Tris hydrochloride (Trise–HCl, #15506017, Invitrogen, Waltham, MA), 150 mM NaCl (Sigma-Aldrich), and 0.1% (v/v) Triton X-100 (Sigma-Aldrich) in DDW.
- Decolorization solution: Dissolve 25% (v/v) Quadrol (#122262, Sigma-Aldrich) and 5% (v/v) ammonium solution (#105432, Sigma-Aldrich) in DDW.
- 30%/50%/70% tert-butanol (tB) gradients (pH > 9.5): Dissolve 30%/50%/70% (v/v) tB (#360538) and 3% (w/v) Quadol in DDW.
- tB-polyethylene glycol (PEG): Dissolve 70% (v/v) tB, 27% (v/v) poly(ethylene glycol) methyl ether methacrylate (PEGMMA)500 (#447943, Sigma-Aldrich), and 3% (w/v) Quadol in DDW.
- Benzyl benzoate (BB)-PEG: Dissolve 75% (v/v) BB (#B6630, Sigma-Aldrich), 25% (v/v) PEGMMA 500 (Sigma-Aldrich), and 3% (w/v) Quadol in DDW.

## 3. Methods

### 3.1. Periapical lesion induction and sample preparation

C57BL/6J mice were subjected to pulp infection. Mice were anesthetized with 62.5 mg/kg ketamine HCl and 12.5 mg/kg xylazine in sterile PBS by intraperitoneal injection. Mandibular first molar pulps were exposed using a No. 1/4 dental round bur (#14820, SS White Dental, Lakewood, NJ) and a variable speed electric handpiece (AEU-178, Aseptico Inc., Woodinville, WA) under a surgical microscope (EVOLUTION XR6; Seiler, St. Louis. MO). All pulp was removed using an .06 C-file (#3670999, Dentsply Sirona, York, PA). The access cavity was left open to facilitate infection of oral bacteria. Animals were euthanized on day 42 after pulp exposure, and the soft tissues around the mandible were carefully removed using a surgical blade. We removed as much soft tissue as possible from the surface of the sample, which is critical to reducing nonspecific background. Then, mandibles were fixed in a fresh 4% PFA solution and decalcified with 15% EDTA for 1 week at room temperature. For histological evaluation, the mandible samples were subjected to paraffin embedding. The embedded mandible samples were sliced at a thickness of 7 μm in buccal-lingual axis. Paraffin sections were deparaffinized with xylene and rehydrated with PBS. Then, to compare the 3D distribution of total and M2 macrophages, the specimens were incubated with an anti-Mac2 antibody (a pan-macrophage marker, 1:500) or an anti-Arg1 antibody (M2 macrophage marker, 1:500) at 4 °C overnight. Using a rabbit-specific HRP/DAB Detection IHC Kit (#ab64261, Abcam), we labeled the primary antibody with streptavidin–biotin, visualized it with a 3,3′-diaminobenzidine substrate in the presence of HRP, and then counterstained with methyl green. For whole-mount staining, the demineralized samples were cut into two sections in the sagittal orientation with a No. 11 surgical blade. Then, the samples were subjected to whole-mount staining.

### 3.2. Whole-mount immunolabeling during the PEGASOS method

Whole-mount immunolabeling was conducted between the decolorization and delipidation steps in the PEGASOS method. For decolorization, samples were pre-washed with DDW for 30 min to elute excess EDTA and subsequently decolored in the decolorization solution for 2 day at 37 °C. After three 1-h washes in TBST, samples were incubated in the blocking buffer for 24 h at room temperature. For macrophage immunolabeling, samples were incubated with a mixture of an anti-Mac2 (1:500) and anti-Arg1 antibodies (1:500) prepared in the staining buffer for 4 day at room temperature. After three washes in TBST, the samples were incubated with a mixture of secondary antibodies, Alexa Fluor 488-conjugated anti-rabbit antibody and Alexa Fluor 594 conjugated anti-rat antibody conjugated, for 4 day at room temperature. After three washes in TBST, the samples were placed in gradient tB delipidation solutions (30%, 50%, 70% v/v tB; each 24 h) for 72 h and then dehydrated in tB-PEG for 2 day at 37 °C. After dehydration, samples were immersed in the BB-PEG medium at 37 °C until the tissue became transparent (typically one day). This series of processes was performed on a shaker at 100 rpm.

Finally, samples were placed in a glass bottom dish (D35-14-1.5N, Cellvis, Mountain View, CA) with BB-PEG medium and covered with cover glass (#12–542A, Thermo Fisher Scientific) for observation. The Stellaris 5 confocal microscope (Leica Microsystems, Buffalo Grove, IL) was employed for capturing fluorescence images and 3D reconstruction. The emission laser we used in this experiment is white laser, and signal detection of Alexa Fluor 488 and Alexa Fluor 594 was achieved by application of FITC and Texas Red filter, respectively. In the sample, a stack of 60 fluorescence images (5 μm thickness, in buccal-lingual direction) were acquired at six different positions in the x–y plane, then the acquired images were stitched, and 3-dimensionally reconstructed using the LASX software (Leica). The Differential interference contrast (DIC) image of cleared mandible sample was captured using a BZ-X700 microscope (Keyence, Itasca, IL) with a Plan Fluor 4x lens and a 0.5 s exposure time.

## 4. Representative results

**Fig. 1.**
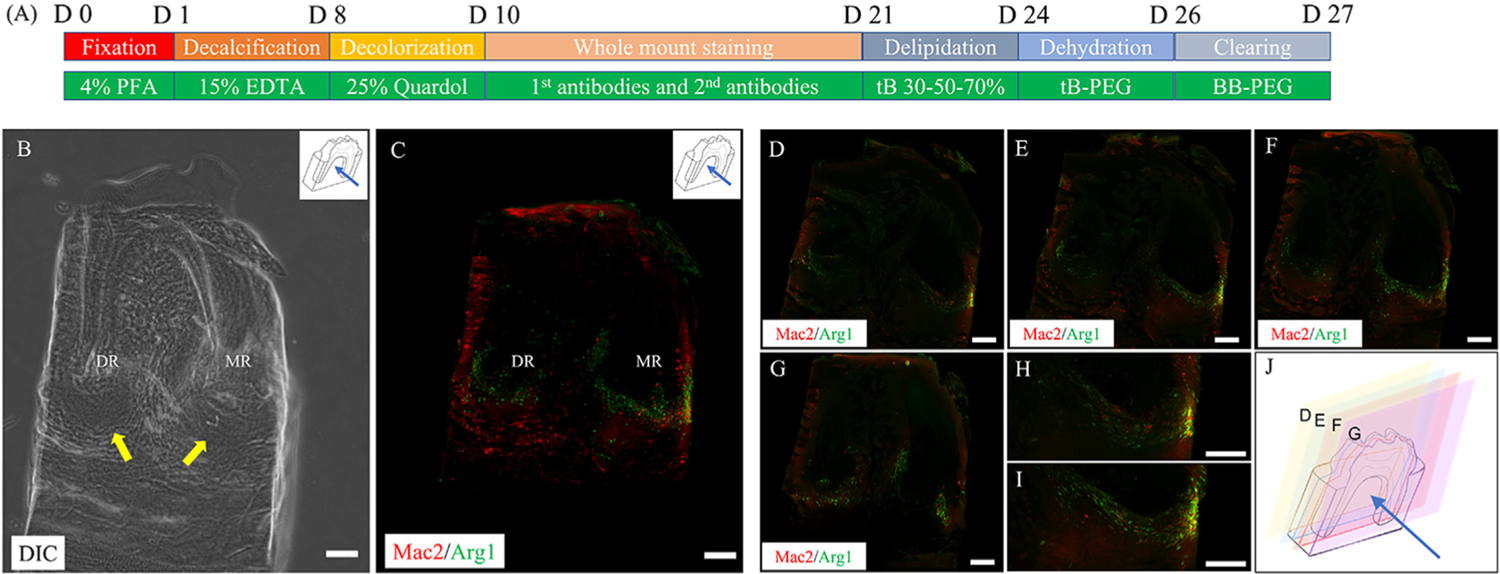
Confocal images of whole-mount immunolabeling of mouse periapical lesions. A bone block containing the periapical lesions surrounding the mesial and distal root apices of the mandibular first molar was harvested and immunolabeled with anti-Mac2 and anti-Arg1 antibodies, followed by tissue clearing using the PEGASOS method. (A) Diagram showing the protocol for whole mount staining and tissue cleaning. The details are described in materials and methods. (B) Differential interference contrast (DIC) image revealed the extent of periapical lesions. (C) 3D distribution of immunolabeled immune cells in periapical lesions shown in a 3D reconstruction image. (D–I) Representative 2D fluorescence images in buccal-lingual direction comprising B. C–F are the 1st, 21st, 41st, and 60th 2D images from the buccal side, respectively. H and I are high magnification of mesial root apex in C and D, respectively. (J) 3D illustration showing where the planes in D–G are located on the cleared mandible sample. Green, Arg1+ cells. Red, Mac2+ cells. DR, Distal root apex. MR, Mesial root apex. Arrow, periapical lesion. Scale bar, 200 μm.

**Fig. 2.**
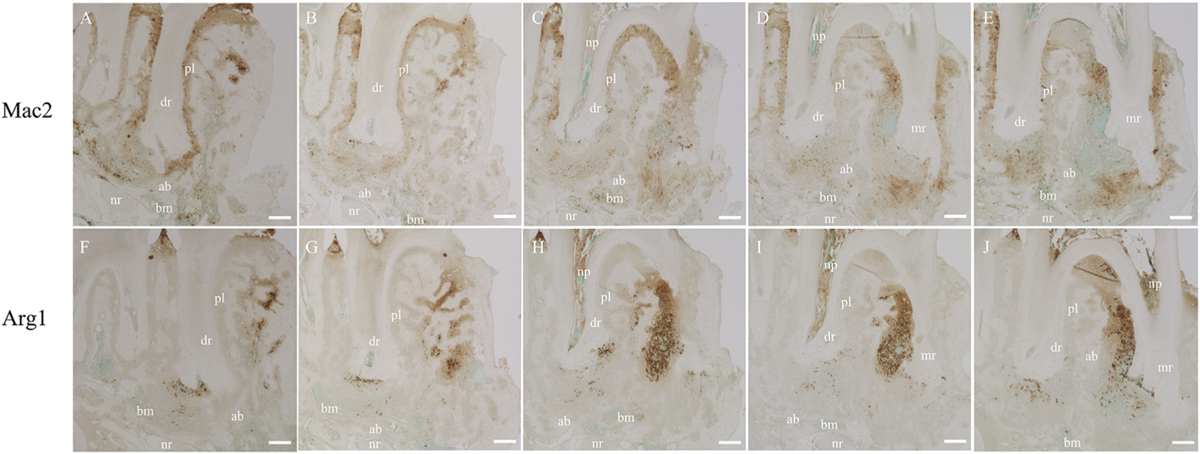
Microscopic images of immunohistochemistry on serial paraffin sections of periapical lesions. A bone block containing the periapical lesions surrounding the mesial and distal root apices of the mandibular first molar was harvested, paraffin-embedded, serially sectioned in the sagittal orientation (buccal to lingual), and immunohistochemically examined with anti-Mac2 and anti-Arg1 antibodies. (A–E) Tissue distribution of Mac2+ cells. (F–J) Tissue distribution of Arg1+ cells. mr, mesial radicular. dr, distal radicular. pl, periodontal ligament. av, alveolar bone. nr, nerve. bm, bone marrow. np, necrotic pulp. Scale bar, 200 μm.

3D visualization revealed that in periapical lesions surrounding the root apex (Fig. 1A), Arg1-positive cells were localized around the root apex, while Mac2-positive cells were widely dispersed inside and outside the lesion (Fig. 1B, Supplementary Fig. 1). On the other hand, as shown in Fig. 2, conventional immunohistochemical sections also showed that Mac2-positive cells were widely distributed in the periapical lesions, periodontal ligament, and bone marrow, while Arg1-positive cells were localized in the periapical region. It is noteworthy that the aspect varies considerably from section to section even within the same sample.

## 5. Discussion

Here, we report a straightforward and effective whole-mount immunolabeling protocol to ascertain the 3D organization and spatial distribution of different immune cell types in mouse periapical lesions. Three-dimensional cell visualization in tooth and mandible samples using transgenic mouse models expressing fluorescent proteins has been reported [10,11], but to our knowledge, there is no report of using whole-mount immunofluorescent labeling for this purpose.

Our method does not require developing genetic mutant models or using specialized techniques, such as serial sectioning. The working time is also shorter than conventional histology. Furthermore, this method could apply to other mineralized tissue samples to monitor the differentiation and dynamics of various cell types, such as osteogenic and odontogenic cells. Despite its utility, this approach demonstrated several limitations. Antibodies validated for conventional immunohistochemistry are not always appropriate for whole-mount immunolabeling, requiring preliminary experiments to determine the suitability of antibodies and staining conditions [12]. In addition, common nuclear staining reagents such as 4,6-diamidino-2-phenylindole dihydrochloride and Hoechst are not applicable to solvent-based clearing methods, including the PEGASOS method. Carbocyanine dyes, including TO-PRO3 and SYTO, should be used [13,14]. Even considering these limitations, the capability to visualize the spatial distribution of the target cells is significant. As shown in Fig. 2, it is difficult to understand the comprehensive cellular distribution in periapical lesions from a conventional histological image without observation in serial sections. A limited number of sections can lead to misinterpretation. The present protocol may provide invaluable insights into dental and craniofacial research.

In conclusion, we developed a technique for whole-mount immunolabeling and optical clearing in rodent mandible samples. This technique enables the visualization of the spatial distribution of multiple immune cell populations in the development of apical periodontitis.

## Supplementary Material

Supplemental data

## Abbreviations:

2D: two-dimensional
3D: three-dimensional
Arg1: arginase 1
BB: benzyl benzoate
DDW: deionized distilled water
EDTA: ethylenediaminetetraacetic acid
NaCl: sodium chloride
PBS: phosphate-buffered saline
PEG: polyethylene glycol
PEGASOS: polyethylene glycol-associated solvent system
PEGMMA: poly(ethylene glycol) methyl ether methacrylate
PFA: paraformaldehyde
tB: tert-butanol
TBST: tris-buffered saline with Triton X-100
Tris–HCl: trizma hydrochloride
